# Relationship between plasma glutamate and cardiovascular disease risk in Chinese patients with type 2 diabetes mellitus by gender

**DOI:** 10.3389/fendo.2023.1095550

**Published:** 2023-04-12

**Authors:** Ru-Tao Li, Yang Li, Bo-Wen Wang, Xiao-Qian Gao, Jing-Xi Zhang, Fan Li, Xiang-Yu Zhang, Zhong-Ze Fang

**Affiliations:** ^1^ School and Hospital of Stomatology, Tianjin Medical University, Tianjin, China; ^2^ Department of Toxicology and Sanitary Chemistry, School of Public Health, Tianjin Medical University, Tianjin, China

**Keywords:** cardiovascular disease, metabolomics, glutamate, type 2 diabetes mellitus, gender

## Abstract

**Objectives:**

This study aimed to assess the association between plasma glutamate (Glu) and the risk of cardiovascular disease (CVD) in patients with type 2 diabetes mellitus (T2DM) and whether this association differs by gender.

**Material and methods:**

We retrieved clinical information on 1032 consecutive patients with T2DM from a same tertiary care center from May 2015 to August 2016. Glu was quantified by liquid chromatography-tandem mass spectrometry analysis. Glu was converted into a categorical variable based on the median concentration in the whole population, while logistic regression was used to obtain the odds ratio (OR) and 95% confidence interval (CI), and the correlation between Glu and various biochemical indices was analyzed.

**Results:**

We found that Glu was positively associated with the risk of CVD in patients with T2DM. This correlation was more significant in women. In T2DM patients, the higher the age, body mass index (BMI), weight and systolic blood pressure (SBP), the lower the glycosylated hemoglobin (HbA1C) concentration and the higher the Glu. In female patients, the correlation between age, weight, BMI, SBP, and plasma Triglycerides (TG), and Glu was also statistically significant.

**Conclusion:**

In conclusion, female T2DM patients with high levels of Glu have a higher risk of developing CVD.

## 1 Introduction

Cardiovascular disease (CVD) is one of the most serious complications of type 2 diabetes mellitus (T2DM), accounting for more than 20% of all-cause deaths in T2DM patients in China (1). In turn, diabetes mellitus patients are also at high risk for CVD (2). Notably, many studies have found that the burden of diabetes varies by gender. For example, 51% of women in Europe have been reported to die from CVD compared to 42% of men (3), with a significant difference between the sexes in this regard. One possible speculation is that men and women may differ in terms of CVD pathology and predictors.

With the development of metabolomics, we have been able to explore the role of a range of metabolites in diseases, including CVD, from a new perspective (4). Metabolomics, the comprehensive analysis of small molecule metabolites in cells, tissues, or whole organisms, has undergone rapid technological advances in the last two decades (5).

Glu homeostasis is essential for various functions such as insulin fraction, gluconeogenesis and glutathione synthesis (6). The role of Glu in metabolic disorders and certain diseases has been explored in several epidemiological studies. The early Framingham Heart Study revealed a positive correlation between Glu levels and insulin resistance in the general population (7).

Previous studies have shown that more than 75% of patients with high Glu levels, especially those with T2DM, die from the cardiovascular-related disease, a figure twice as high as that of Non-diabetes mellitus (8). A German cross-sectional study found an association between Glu levels and higher CVD (9). A case-control study in China also suggests this idea (10).Although several studies have described the relationship between glutamate and CVD, it is still lacking whether there is some association between CVD and Glu in patients with type 2 diabetes.

In this study, we established a cross-sectional study in a Chinese population with the aim of exploring the relationship between plasma Glu and CVD risk in patients with type 2 diabetes and to examine whether this relationship differs by gender.

## 2 Methods

### 2.1 Study populations

The First Affiliated Hospital of Liaoning Medical University in Jinzhou, Liaoning Province, a tertiary care center, established a metabolomics laboratory in 2013 to provide metabolomics testing to outpatients and inpatients or individuals who agreed to pay for a physical examination.

Inclusion criteria were 1. Diagnosed with T2DM according to the 1999 World Health Organization criteria (11). 2. On diabetes medication. The exclusion criteria were: 1. Under the age of 18 years. 2. Living in the hospital’s service area as a local resident for less than six months prior to the start of the study. 3. Diabetes secondary to other diseases. 4. Having a mental illness that makes it difficult to cooperate with health screening. Among consecutive patients aged 18 years or older with complete data on height, weight, and blood pressure, 1032 cases were diagnosed with T2DM, of whom those suffering from stroke or myocardial infarction, or both, we designated them as the case group, and the rest of the T2DM patients we designated as the control group.

The Clinical Research Committee of the First Affiliated Hospital of Liaoning Medical University approved the ethical nature of this study, and informed consent was waived due to the retrospective nature of this study, following the Declaration of Helsinki.

### 2.2 Data collection and definitions

Retrieved data for these cases included demographic and anthropometric information, as well as current clinical parameters, medications, and complications of diabetes. Clinical parameters included glycated hemoglobin, blood pressure and lipids. Diabetic complications included coronary heart disease, cerebrovascular disease, diabetic retinopathy, and diabetic nephropathy. We documented detailed medication use, including oral antidiabetic drugs (OADs) and insulin, angiotensin-converting enzyme inhibitors (ACEIs), angiotensin receptor blockers (ARBs), and other antihypertensives, statins, and other lipid-lowering drugs.

CVD was defined as a history of coronary heart disease or stroke. Coronary heart disease was defined as a history of angina pectoris, abnormal ECG or stress test, myocardial infarction, angina pectoris, coronary artery bypass grafting, or angioplasty; stroke was defined as non-fatal subarachnoid hemorrhage, cerebral hemorrhage or other unspecified intracranial hemorrhage and ischemic stroke. Body mass index (BMI) was calculated as weight in kilograms divided by height in meters squared.

### 2.3 Laboratory tests

Capillary whole blood was collected after fasting for at least 8 hours and preserved as dried blood spots for metabolomic analysis. Metabolites in dried blood spots were determined by direct infusion mass spectrometry using an AB Sciex 4000 QTrap system (AB Sciex, Framingham, MA, USA). High-purity water and acetonitrile from Thermo Fisher (Waltham, MA, USA) were used as diluent and mobile phases. 1-Butanol and acetyl chloride from Sigma-Aldrich (StLouis, MO, USA) were used to obtain samples. Isotopically labeled internal standard samples of 12 amino acids (NSK-A) were purchased from Cambridge Isotope Laboratories (Tewksbury, MA, USA), while standard samples of amino acids were purchased from Chrom Systems (Grafelfing, Germany).

### 2.4 Statistical analysis

For missing CVD, case deletion was performed. And for missing other biochemical indices, multiple interpolation was used to fill in the missing values. Normality was tested by Q-Q plots or P-P plots. The quantitative data for normal distributions were expressed as mean ± standard deviation (SD), and the data that did not obey the normal distribution were expressed as the median interquartile range (IQR). The continuous variable was judged by student’s t-test or Wilcox-W test when appropriate.

Categorical data were expressed as n (%), and X^2^ tests (or Fisher test, if applicable) were used to compare the differences in categorical variables between the CVD and non-CVD groups.

Binary logistic regression models were used to obtain the odd ratio (OR) and 95% confidence interval (CI). A multivariable model was used to adjust for the confounding effects of other variables. The unadjusted OR was first obtained, and then a multivariable analysis was performed to include confounding factors including age, BMI, smoking, alcohol consumption, systolic blood pressure (SBP), glycated hemoglobin (HbA1C), high-density lipoprotein cholesterol(HDL-C), low-density lipoprotein cholesterol (LDL-C), Triglyceride(TG), and medication use to obtain a structured adjusted OR.

To explore a possible nonlinear association between Glu and CVD, we used the median as the cutoff point in logistic regressions. The Glu was divided into two segments according to the median, and the relationship between Glu and CVD in different gender groups was analyzed in the T2DM patient population to obtain the corresponding OR and 95% CI.

Pearson or Spearman correlations were used to calculate the correlation coefficients within Glu and clinical biochemical parameters, namely age, SBP, DBP, BMI, height, weight, TG, HbA1, HDL-C, and LDL-C.

IBM SPSS Statistics was used for statistical analysis, and linear plots for correlation analysis were drawn using RStudio 4.0.5. All P values were two-tailed, and P < 0.05 was considered a statistically significant difference.

## 3 Results

### 3.1 Characteristics of the study population

**Table 1** demonstrates the basic characteristics of the all people as well as the different gender groups. In all people, patients with CVD were older, had higher SBP, higher glutamate concentrations, lower HbA1C, lower LDL-C concentrations, less use of insulin, statins, ARBs, and more use of ACEI drugs than those without CVD. In women, patients with CVD were older than those without CVD, had higher systolic blood pressure, used statins more often, and other antihypertensive drugs, used ACEI and ARB drugs less often, and whether they smoked and drank alcohol, used insulin, other hypoglycemic drugs, other lipid-lowering drugs, weight, height, BMI, DBP, Glu, TG, HDL-C, or LDL-C were no significant difference. In men, patients with CVD were older, heavier, and lower in height, had higher Glu, lower HbA1C, lower LDL-C, used ACEI and other antihypertensive drugs more often, used insulin, other hypoglycemic drugs, statins less often, had alcohol habits, smoked or not, used or not other lipid-lowering drugs, BMI, diastolic blood pressure, TG, HDL-C, and LDL-C than patients without CVD. TG, HDL-C were not significantly different.

**Table 1:** Clinical and biochemical characteristics of participants according to the occurrence of CVD.

Variables	All people (N=1032)			Women (N=483)			Men (N=549)		
	CVD	Non-CVD	P-Value	CVD	Non-CVD	P-Value	CVD	Non-CVD	P-Value
Numbers	350 (33.9%)	682 (66.1%)		160 (33.1%)	323 (66.9%)		190 (34.6%)	359 (65.4%)	
Smoking	104 (10.1%)	227 (22.0%)	0.245	10 (2.1%)	23 (4.8%)	0.721	94 (17.1%)	204 (37.2%)	0.100
Drinking	88 (8.5%)	202 (19.6%)	0.130	11 (2.3%)	4 (0.8%)	0.589	84 (16.0%)	191 (34.8%)	0.045
Age (years)	64.78 ± 11	53.37 ± 13.52	<0.001	65.21 ± 9.79	56.56 ± 12.42	<0.001	64.42 ± 11.94	50.49 ± 13.83	<0.001
Weight (kg)	69.326 ± 11.8	70.862 ± 13.8	0.062	63.94 ± 10.12	64.48 ± 11.69	0.617	73.86 ± 11.20	76.60 ± 13.02	0.010
Height (cm)	166 (160, 172)	167 (160, 173)	0.177	160 (156, 163)	160 (158, 164)	0.118	171.34 ± 5.98	172.38 ± 5.66	0.045
BMI (kg/m2)	25.16 ± 3.74	25.36 ± 3.91	0.423	25.17 ± 3.89	24.96 ± 3.98	0.59	25.15 ± 3.61	25.722 ± 3.82	0.090
SBP,mmHg	145.79 ± 25.45	137.65 ± 22.73	<0.001	145.12 ± 27.02	139.94 ± 24.71	0.036	146.35 ± 20.62	135.60 ± 20.62	<0.001
DBP,mmHg	83.21 ± 15.48	82.07 ± 12.37	0.231	82.29 ± 15.33	80.74 ± 12.28	0.268	83.99 ± 15.60	83.26 ± 12.38	0.577
Glu,μmol/L	103.63 ± 33.34	105.85 ± 39.43	0.012	109.17 ± 37.11	104.20 ± 40.48	0.193	108.20 ± 34.02	101.21 ± 32.76	0.019
HbA1c,%	9.28 ± 2.38	9.78 ± 2.35	0.001	9.38 ± 2.50	9.75 ± 2.42	0.097	9.21 ± 2.28	9.82 ± 2.28	0.003
TG,mmol/L	1.65 (1.13,2.22)	1.73 (1.15,2.48)	0.065	1.71 (1.14,2.36)	1.73 (1.20, 2.47)	0.528	1.58 (1.10,2.16)	1.70 (1.13,2.50)	0.060
HDL-C,mmol/L	1.02(0.83,1.26)	1.01 (0.85,1.24)	0.999	1.10 (0.91,1.28)	1.06 (0.87, 1.26)	0.413	0.94 (0.80,1.20)	0.96 (0.83,1.23)	0.495
LDL-C,mmol/L	2.77 (2.13,3.31)	2.84 (2.32,3.44)	0.022	2.87 (2.34,3.40)	2.92 (2.31, 3.49)	0.376	2.71 (2.06,3.19)	2.78 (2.33,3.35)	0.020
Insulin	237 (23.0%)	535 (51.8%)	<0.001	115 (23.8%)	245 (50.7%)	0.345	122 (22.2%)	290 (52.8%)	<0.001
Other hypoglycemic drugs	182 (17.6%)	387 (37.5%)	0.147	92 (19.0%)	179 (37.1%)	0.664	90 (16.4%)	208 (37.9%)	0.018
Statins	184 (17.8%)	186 (18.0%)	<0.001	88(18.2%)	88(18.2%)	<0.001	96(17.5%)	98(17.9%)	<0.001
Other lipid-lowering drugs	5 (0.5%)	18 (1.7%)	0.212	3(0.6%)	4(0.8%)	0.582	2(0.4%)	14(2.6%)	0.059
ACEIs	68 (6.6%)	67 (6.5%)	<0.001	30 (6.2%)	34 (7.0%)	0.012	38 (6.9%)	33 (6.0%)	<0.001
ARBs	64 (6.2%)	70 (6.8%)	<0.001	34 (7.0%)	45 (9.3%)	0.041	30 (5.5%)	25 (4.6%)	0.001
Other antihypertensives	178 (17.2%)	131 12.7%)	<0.001	77 (15.9%)	75 (15.5%)	<0.001	101 (18.4%)	56 (10.2%)	<0.001

BMI, body mass index; SBP, systolic blood pressure, DBP, diastolic blood pressure; Glu, glutamate;HbA1c, glycated hemoglobin; TG, Triglyceride; HDL-C, high density lipoprotein cholesterol; LDL-C, low-density lipoprotein cholesterol; ACEIs,angiotensin-converting enzyme inhibitors; ARBs angiotensin receptor blockers.

Data are mean ± standard deviation, median (IQR), or n (%).

P values were derived from the t-test for normally distributed variables, Mann-Whitney U test for skewed distributions, Chi-square test for categorical variables. P < 0.05 was defined as statistically significant.

### 3.2 Association of Glu with CVD risk in patients with T2DM

In all patients, after standardization of Glu, unadjusted OR=1.176, p=0.012 was obtained using univariable model binary logistic regression. Glu was positively associated with the risk of CVD prevalence (OR=1.176,95% CI:1.036,1.334).

Age, BMI, smoking, alcohol consumption, SBP, HbA1C, HDL-C, LDL-C, TG, and medication use were included as confounders in a multivariable model for analysis, and this correlation was found to be no longer statistically significant, yielding an adjusted OR=1.003, p=0.105. The adjusted positive correlation was attenuated (OR=1.003, 95% CI:0.999,1.008). In the male group, using univariable model, an unadjusted OR=1.006, p=0.021; adjusted OR=1.005, p=0.139. In the female group, using univariable model, an unadjusted OR=1.003, p=0.194; adjusted OR=1.003, p=0.318. The relationship between Glu and CVD was not statistically significantly different in the multivariable model in both men and women. The results are shown in **Table 2**.

**Table 2:** Relationship between Glu and CVD in patients with type 2 diabetes.

Glu (μmol/L)	OR	95%CI	*P* Value
Univariable model			
All people	1.176	1.036-1.334	0.012
Men	1.006	1.001-1.012	0.021
Women	1.003	0.998-1.008	0.194
Multivariable model^a^			
All people	1.003	0.999-1.008	0.105
Men	1.005	0.998-1.012	0.139
Women	1.003	0.997-1.008	0.318

Glu, glutamate; OR: odds ratio; CI, confidence interval.

a Model adjusted for age, BMI, smoking, alcohol consumption, SBP, HbA1C, HDL-C, LDL-C, TG, and medication use.

### 3.3 Interaction between Glu and gender

Logistic regression was performed by converting Glu to categorical variables according to the median. Higher Glu increased the risk of CVD in patients with T2DM (OR: 1.657, 95% CI, 1.277-2.150, P<0.001, adjusted OR: 1.539, 95% CI, 1.156-2.050, P=0.003). In the male group, the unadjusted OR= 1.580 (95% CI, 1.107-2.253), P=0.012, and the adjusted OR= 1.552 (95% CI, 0.989-2.433), P=0.056, with no significant correlation between Glu and risk of CVD. In the female group, the unadjusted OR= 1.747, (95% CI 1.191-2.563), P=0.004, and the adjusted OR = 1.591, (95% CI 1.024-2.471), P=0.039, higher Glu was associated with increased risk values for CVD in patients with T2DM. The results are shown in **Table 3**.

**Table 3:** Association between dichotomized Glu and CVD risk in patients with type 2 diabetes.

Glu≥97.9vsGlu<97.9 (μmol/L)	OR	95%CI	*P* Value
Univariable model			
All people	1.657	1.277-2.150	<0.001
Men	1.580	1.107-2.253	0.012
Women	1.747	1.191-2.563	0.004
Multivariable model^a^			
All people	1.539	1.156-2.050	0.003
Men	1.552	0.989-2.433	0.056
Women	1.591	1.024-2.471	0.039

Glu, glutamate; OR: odds ratio; CI, confidence interval.

a Model adjusted for age, BMI, smoking, alcohol consumption, SBP, HbA1C, HDL-C, LDL-C, TG, and medication use.

### 3.4 Correlation of Glu with clinical biochemical parameters

**Figure 1:**
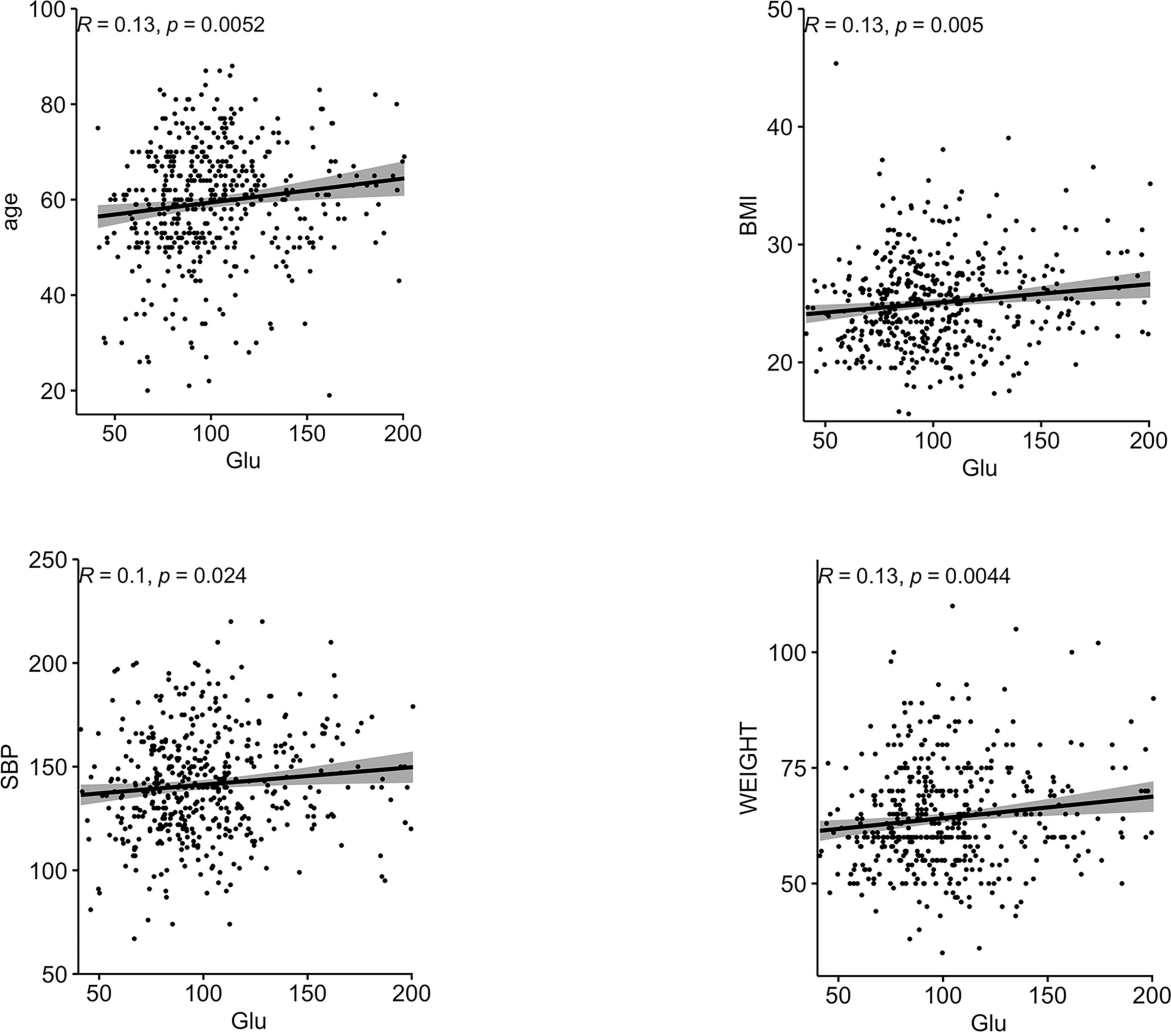
Correlation between Glu and each biochemical index in all people. BMI, body mass index; HbA1c, glycated hemoglobin; SBP, systolic blood pressure; Glu, glutamate.

**Figure 2:**
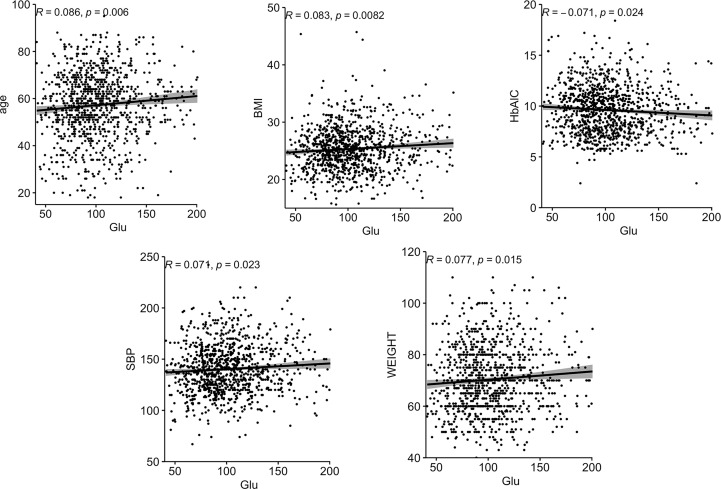
Correlation between Glu and various biochemical indicators in the female group. BMI, body mass index; SBP, systolic blood pressure; Glu, glutamate.

Correlations between Glu and various biochemical parameters were calculated using Pearson correlation coefficients. Among all patients with T2DM, the relationship between them is shown in **Figure 1**.The older the age, the higher the BMI, the higher the SBP, the heavier the weight, the lower the HbA1C, and the higher the Glu. In the female group, the relationship between them is shown in **Figure 2**, the older the age, the higher the BMI, the higher the SBP and the heavier the weight, the higher the Glu. Correlations with other biochemical indicators are plotted in the **Supplementary Material**.

## 4 Discussion

In this study, we found that plasma Glu levels above the population median concentration increased the risk of CVD in patients with type 2 diabetes, and this relationship remained significant in women with type 2 diabetes.

Some data analyzing the correlation between Glu receptor genes and angiogenic genes suggest that Glu receptors have anti-vascular effects, that the expression of genes controlling the production of this receptor is negatively correlated with angiogenic genes, and that changes in Glu receptor activity may affect the formation of microvascular networks (12). Glu ionotropic receptors are present in the center and periphery, and peripheral ones are also present in pancreatic islet cells (13). It was demonstrated that the activation of the receptor by elevated Glu and its dual action on islet cells may exacerbate the destruction of vascular endothelial cells, leading to an increased likelihood of CVD in diabetic patients. It has also been shown that such receptors are also present in the heart and blood vessels (14). Activation of the receptor promotes calcium inward flow, which in the cardiovascular system determines excitation-contraction coupling, and increasing Glu may lead to excessive activation of these receptors, resulting in intracellular calcium overload in cardiac myocytes, which in turn leads to apoptosis, which may be one of the pathogenic mechanisms (15). Also some studies on glutamate proved that it can stimulate the release of glucagon in pancreatic alpha cells (16) and increase the transamination of pyruvate to alanine, a powerful promoter of the abundant gluconeogenesis in obese patients (17).Glutamate is also known as a direct precursor of alpha-ketoglutarate, an intermediate in the Krebs cycle, which acts as an anabolic and anti-catabolic source of energy for many cell types (18). In our study, higher levels of Glu in plasma increased the risk of CVD in women with type 2 diabetes, but not in men. Although women’s estrogen levels are higher than men’s, which is important for maintaining vascular health and promoting blood vessel growth (19), the decline in estrogen levels in women after menopause greatly weakens this protective effect, and the average age of female participants in this study is higher than the age at which women develop menopause. Therefore, we hypothesized that in the presence of elevated Glu, which tend to destroy the vascular endothelium, women with T2DM are more susceptible to its negative effects, thus increasing the risk of CVD.

When Glu was used as a continuous variable, no significant relationship was found with CVD in T2DM, while when Glu was transformed into a categorical variable according to median concentration, higher levels of plasma Glu were positively associated with CVD in all T2DM patients and in female T2DM patients. Once Glu is elevated to a certain threshold, it will cause cardiovascular abnormalities in the body. For example, it has been found that glutamate has a positive correlation with BMI, waist circumference, glucose, insulin, insulin resistance index, systolic blood pressure, diastolic blood pressure and triglycerides, and a negative correlation with HDL-C (7).

Our study has important implications for public health. The diabetes epidemic poses a major health and economic threat to the world, including China. Early management of its important co-morbidities with lifestyle and medical interventions is essential. Our findings provide new predictive ideas for myocardial infarction and ischemic stroke prevention in T2DM patients, especially for female patients. This provides a new direction for further research into the possible role of Glu in T2DM patients suffering from CVD.

There are some limitations of this study. First, due to the nature of retrospective cross-sectional studies, these findings cannot determine the causal relationship between Glu and diabetic CVD, and a larger study in a population is needed. Also, this study was limited to Chinese patients and is not representative of other countries and ethnicities. In addition, the exact level of Glu above which it significantly increases the risk of CVD in female patients with T2DM needs to be studied in more depth. Finally, although some of the confounding factors were screened and taken into account in this study, the effect of other potential confounding factors on the results cannot yet be completely eliminated.

In conclusion, we found that higher plasma concentrations of Glu were associated with an increased risk of CVD in patients with T2DM and that this association remained significant in female patients, but not in male patients. Therefore, this result is derived from a cross-sectional study, so further prospective studies and mechanistic studies are needed to further validate this conclusion.

## Data availability statement

The raw data supporting the conclusions of this article will be made available by the authors, without undue reservation.

## Author contributions

R-TL wrote the article. YL and B-WW analyzed the data for this article. X-QG and J-XZ collected and collated the data. FL reviewed the relevant literature. Z-ZF and X-YZ conceived the project and designed the experiments. All authors contributed to the article and approved the submitted version.
