# Supplementary material for: Relationship between plasma glutamate and cardiovascular disease risk in Chinese patients with type 2 diabetes mellitus by gender
Source: Front Endocrinol (Lausanne). 2023 Apr 12;14:1095550. doi: 10.3389/fendo.2023.1095550 (PMC10130405; doi:10.3389/fendo.2023.1095550)
Supplement: Supplementary file 1 [file DataSheet_1.docx]

Supplementary Material

# Supplementary Figures


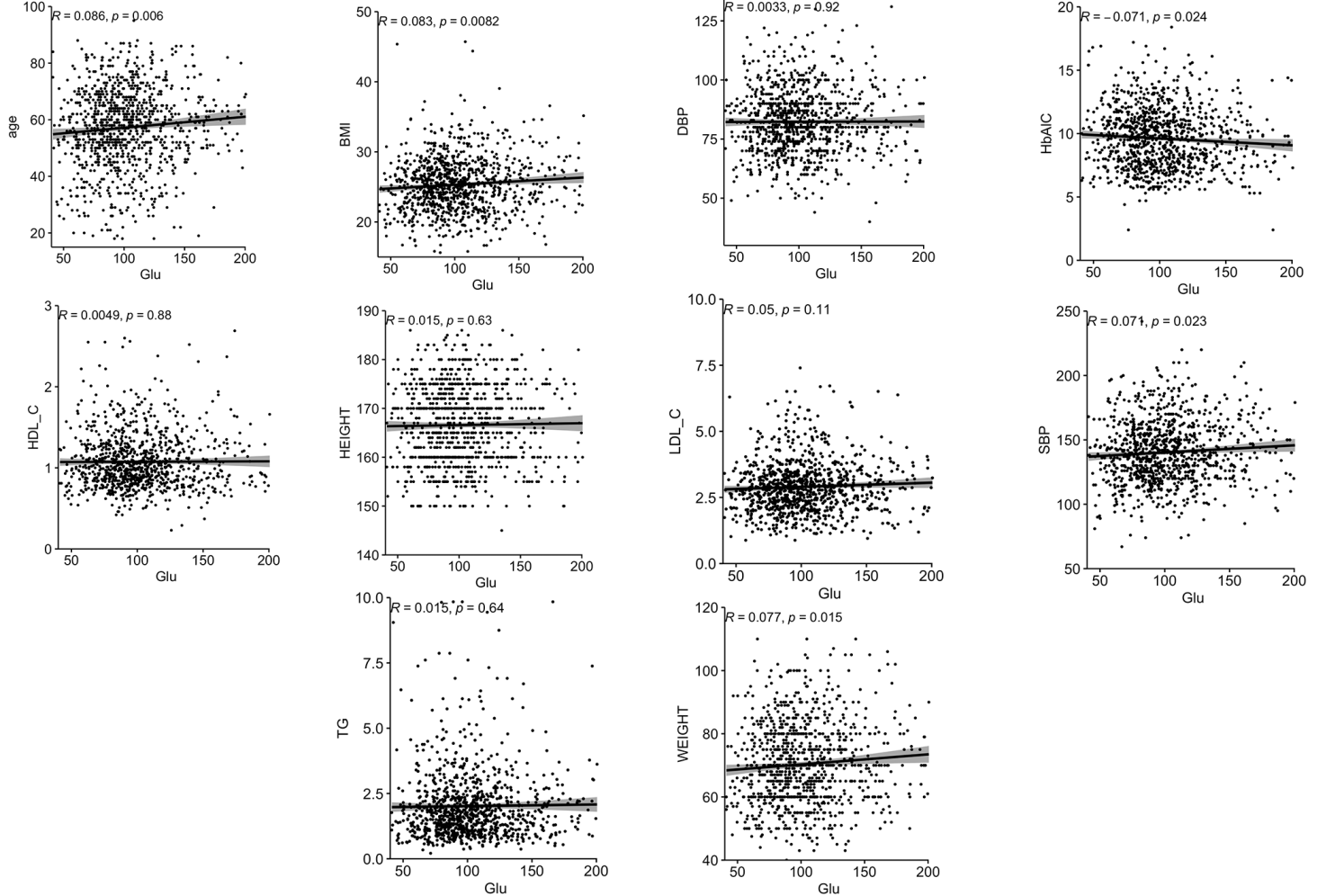


**Supplementary Figure 1**  Correlation between Glu and each biochemical index in all people. BMI, body mass index; DBP, diastolic blood pressure; HbA1c, glycated hemoglobin; HDL-C, high density lipoprotein cholesterol; LDL-C, low-density lipoprotein cholesterol; SBP, systolic blood pressure; TG, Triglyceride; Glu, glutamate.


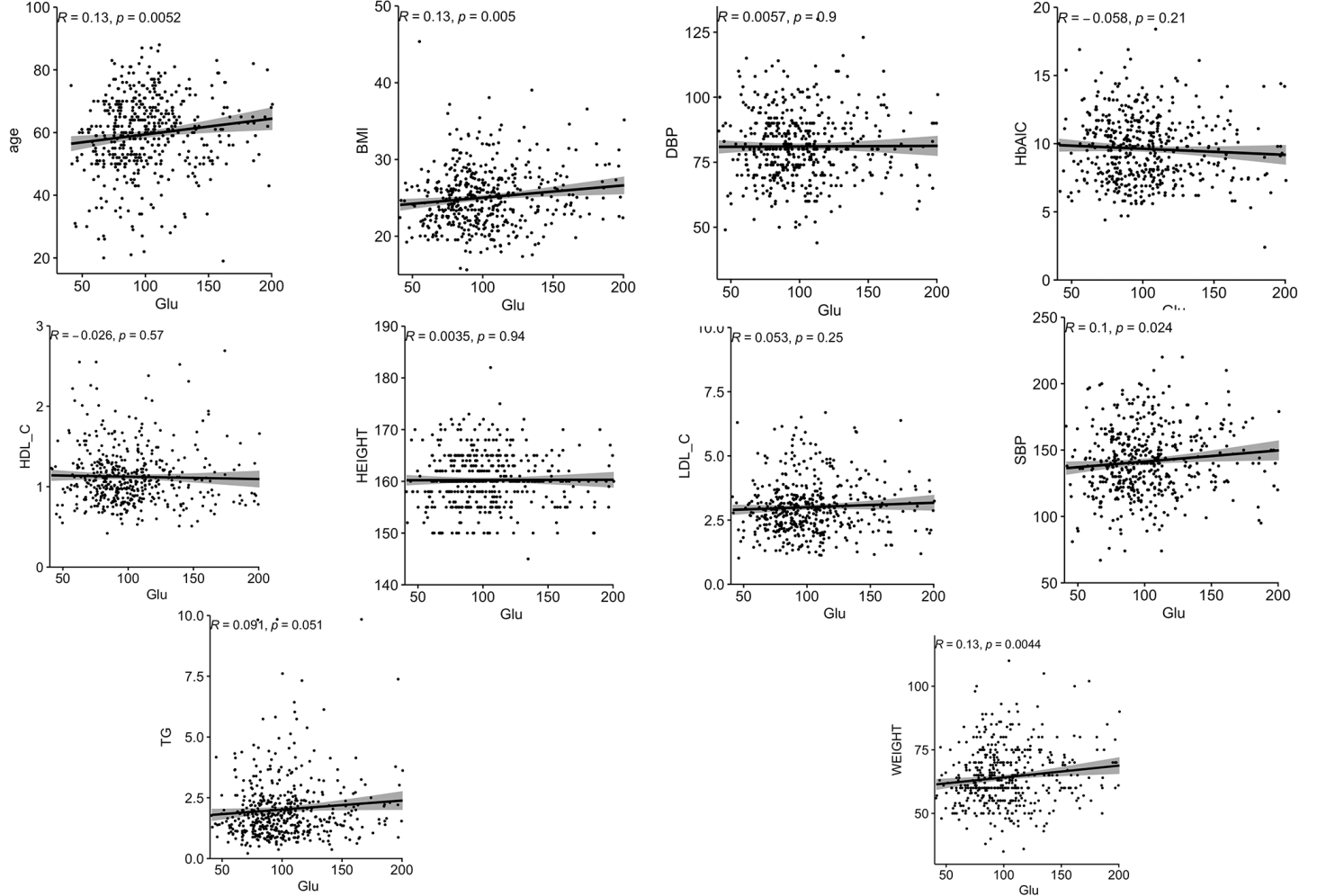


**Supplementary Figure 2**  Correlation between Glu and each biochemical index in women. BMI, body mass index; DBP, diastolic blood pressure; HbA1c, glycated hemoglobin; HDL-C, high density lipoprotein cholesterol; LDL-C, low-density lipoprotein cholesterol; SBP, systolic blood pressure; TG, Triglyceride; Glu, glutamate.


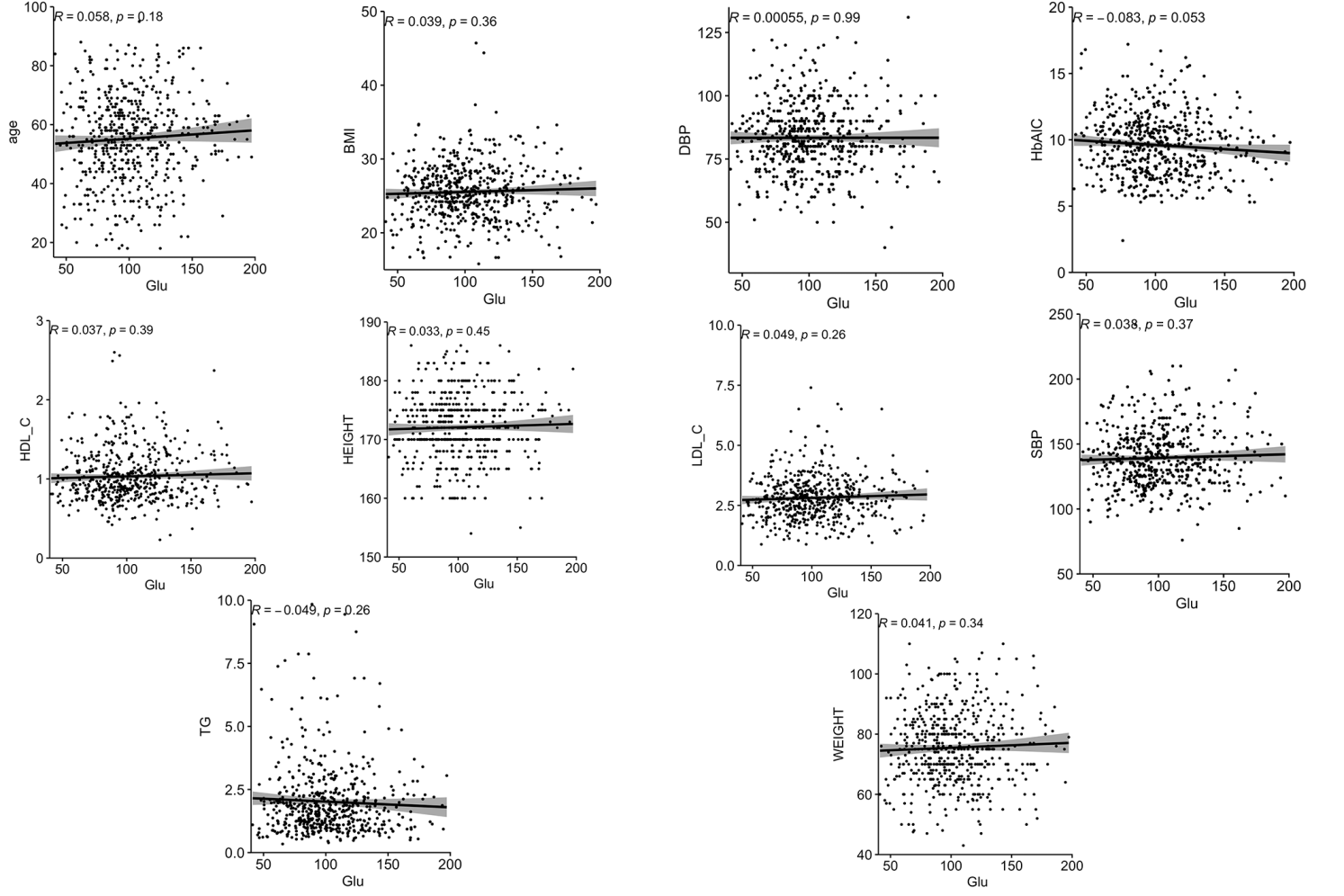


**Supplementary Figure 3**  Correlation between Glu and each biochemical index in men. BMI, body mass index; DBP, diastolic blood pressure; HbA1c, glycated hemoglobin; HDL-C, high density lipoprotein cholesterol; LDL-C, low-density lipoprotein cholesterol; SBP, systolic blood pressure; TG, Triglyceride; Glu, glutamate.
